# 9,9-Dimethyl-12-(3-nitro­phen­yl)-7,8,9,10,11,12-hexa­hydro­benz[*a*]acridin-11-one

**DOI:** 10.1107/S1600536809033935

**Published:** 2009-08-29

**Authors:** Runhong Jia, Juhua Peng, Shujiang Tu

**Affiliations:** a Lianyungang Teacher’s College, Lianyungang 222006, People’s Republic of China; b College of Chemistry and Chemical Engineering, Xuzhou Normal University, Xuzhou 221116, People’s Republic of China

## Abstract

The title compound, C_25_H_22_N_2_O_3_, was synthesized by the reaction of 3-nitro­benzaldehyde, dimedone and 2-naphthyl­amine in ethanol. In the mol­ecular structure, the cyclo­hexenone ring adopts an envelope conformation, whereas the piperidine ring has a boat conformation. The crystal packing is stabilized by inter­molecular N—H⋯O hydrogen bonds.

## Related literature

For the biological and physical activity of compounds containing the acridine skeleton, see: Wysocka-Skrzela & Ledochowski (1976 ▶); Matsumoto *et al.* (1983 ▶); Popielarz *et al.* (1997 ▶). Jia *et al.* (2007 ▶); Kidwai & Rastogi (2005 ▶); Srividya *et al.* (1996 ▶). For microwave irradiation in organic synthesis, see: Tu *et al.* (2002 ▶, 2004 ▶). For related structures, see: Jia *et al.* (2006 ▶); Wang *et al.* (2006 ▶); Tu *et al.* (2006 ▶).
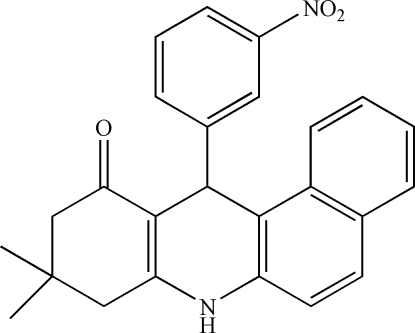


## Experimental

### 

#### Crystal data

C_25_H_22_N_2_O_3_
*M* *_r_* = 398.45
Monoclinic, 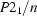

*a* = 10.264 (7) Å
*b* = 13.099 (9) Å
*c* = 15.018 (10) Å
β = 94.403 (10)°
*V* = 2013 (2) Å^3^
*Z* = 4
Mo *K*α radiation
μ = 0.09 mm^−1^
*T* = 298 K
0.17 × 0.16 × 0.10 mm

#### Data collection

Bruker SMART CCD area-detector diffractometer
Absorption correction: multi-scan (*SADABS*; Bruker, 1998 ▶) *T*_min_ = 0.985, *T*_max_ = 0.991
10293 measured reflections
3541 independent reflections
1401 reflections with *I* > 2σ(*I*)
*R*_int_ = 0.078

#### Refinement

*R*[*F* ^2^ > 2σ(*F* ^2^)] = 0.055
*wR*(*F* ^2^) = 0.185
*S* = 1.01
3541 reflections
273 parameters
H-atom parameters constrained
Δρ_max_ = 0.18 e Å^−3^
Δρ_min_ = −0.19 e Å^−3^

### 

Data collection: *SMART* (Bruker, 1998 ▶); cell refinement: *SAINT* (Bruker, 1998 ▶); data reduction: *SAINT*; program(s) used to solve structure: *SHELXS97* (Sheldrick, 2008 ▶); program(s) used to refine structure: *SHELXL97* (Sheldrick, 2008 ▶); molecular graphics: *SHELXTL* (Sheldrick, 2008 ▶); software used to prepare material for publication: *SHELXTL*.

## Supplementary Material

Crystal structure: contains datablocks global, I. DOI: 10.1107/S1600536809033935/zq2005sup1.cif

Structure factors: contains datablocks I. DOI: 10.1107/S1600536809033935/zq2005Isup2.hkl

Additional supplementary materials:  crystallographic information; 3D view; checkCIF report

## Figures and Tables

**Table 1:** Hydrogen-bond geometry (Å, °)

*D*—H⋯*A*	*D*—H	H⋯*A*	*D*⋯*A*	*D*—H⋯*A*
N1—H1⋯O1^i^	0.86	2.13	2.937 (4)	156

Symmetry code: (i) 
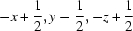
.

**Fig. 1.**
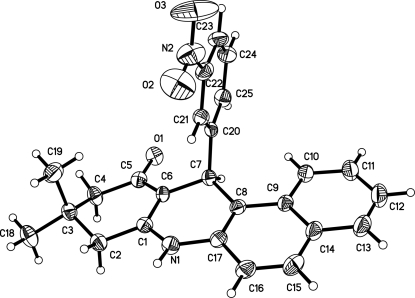
The molecular structure of the title compound, showing 30% probability displacement ellipsoids.

**Fig. 2.**
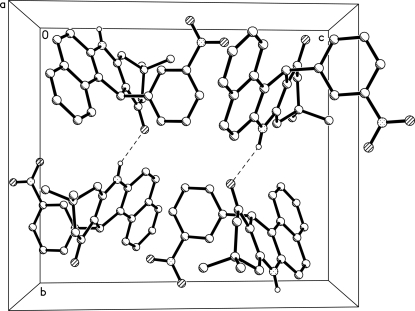
The packing diagram of the title compound viewed along the a axis.

## supplementary crystallographic information

### Comment

A lot of natural and synthetic compounds containing the acridine skeleton display interesting biological and physical activities, such as antimalaria (Wysocka-Skrzela *et al.* 1976) and antitumor agents (Matsumoto *et al.* 1983), and multihydroacridineone derivatives have been reported to have high fluorescence efficiency and can be used as fluorescent molecular probes for monitoring of polymerization process (Popielarz *et al.* 1997). They are also increasingly receiving attention due to their likeness in properties with those of 1,4-dihydropyridines, which have similarities in structure to the biologically important compounds such as NADH and NADPH (Srividya *et al.* 1996). As a consequence, the interest of organic chemists in the synthesis or structure modifications of acridinedione derivatives remains high. Microwave irradiation is a very useful technique in organic synthesis (Tu *et al.* 2002). It is a simple, timesaving, high yielding, and environmentally friendly process. We have already reported the synthesis of heterocyclic compounds under microwave irradiation (Tu *et al.* 2004). M. Kidwai and S. Rastogi have reported the synthesis of polyhydroacridinones without additional fused benzene rings (Kidwai *et al.* 2005). The efficiency of microwave irradiation in promoting organic reaction and the success of its application in heterocyclic synthesis (Jia *et al.* 2007) has rapidly gained in popularity. Therefore design and synthesis of these compounds has been challenging. For these reasons, the synthesis of compounds containing cyanopyridine derivatives is strongly desired. In this paper we report the crystal structure of the title compound (I).

In the crystal structure, the dihedral angle between the C1/C6/C8/C17/N1 plane and the C20—C25 benzene ring is 83.5 (8)° (Fig. 1). The dihedral angle between the C1/C6/C8/C17/N1 plane and the C8/C9/C14/C15/C16/C17 plane is 5.8 (5)°. The dihedral angle between the C1/C6/C8/C17/N1 plane and the C1/C2/C4/C5/C6 plane is 11.4 (4)°, indicating that they are almost parallel. The distance between atom C3 and the mean plane C4/C5/C6/C1/C2 is 0.594 (6) Å, showing that the cyclohexenone ring adopts an envelope conformation. The newly formed piperidine ring has a boat conformation with the atoms N1 and C7 deviating from the plane C1/C6/C17/C8 by 0.098 (5)  and 0.184 (6) Å, respectively, on the same direction. One chiral center exists in the title compound at C7. The centrosymmetric space group indicates the presence of equimolar enantiomers (S and R) in the crystal structure. The molecules are connected *via* N1—H1···O1 hydrogen bonds, forming infinite one-dimensional chains along the *b* axis (Fig. 2).

### Experimental

Compound (I) was prepared by the reaction of 3-nitrobenzaldehyde (1 mmol), dimedone (1 mmol), 2-naphthylamine (1 mmol), in ethanol (2 ml) under microwave irradiation without catalyst. Single crystals of (I) suitable for X-ray diffraction were obtained by slow evaporation of a 95% aqueous ethanol solution (yield 92%; m.p. 567–569 K). IR (cm^-1^): 3260, 2928, 1582, 1522, 1494, 1385, 1234, 1155, 1093, 982, 923, 811, 729, 687, 654; ^1^H NMR (DMSO-d_6_): 0.82 (s, 3H, CH3), 1.06 (s, 3H, CH3), 2.04 (d, 1H, J = 16.0 Hz, CH), 2.25 (d, 1H, J = 16.0 Hz, CH), 2.43 (d, 1H, J = 16.4 Hz, CH), 2.58 (d, 1H, J = 16.4 Hz, CH), 5.97 (s, 1H, CH), 7.48–7.31 (m, 4H, ArH), 7.70 (d, 1H, J = 7.6 Hz, ArH),7.96–7.82 (m, 4H, ArH), 8.07 (s, 1H, ArH), 9.90 (s, 1H, NH).

### Refinement

All H atoms were positioned geometrically and treated as riding, with N—H = 0.86 Å and C—H = 0.93–0.97 Å, and with U_iso_(H) = 1.5Ueq(C) for methyl H atoms and U_iso_(H) = 1.2Ueq(C,N) for others.

### Crystal data

**Table:**

C_25_H_22_N_2_O_3_	*F*(000) = 840
*M**_r_* = 398.45	*D*_x_ = 1.315 Mg m^−^^3^
Monoclinic, *P*2_1_/*n*	Melting point = 567–569 K
Hall symbol: -P 2yn	Mo *K*α radiation, λ = 0.71073 Å
*a* = 10.264 (7) Å	Cell parameters from 1084 reflections
*b* = 13.099 (9) Å	θ = 2.5–22.0°
*c* = 15.018 (10) Å	µ = 0.09 mm^−^^1^
β = 94.403 (10)°	*T* = 298 K
*V* = 2013 (2) Å^3^	Block, colourless
*Z* = 4	0.17 × 0.16 × 0.10 mm

### Data collection

**Table:**

Bruker SMART CCD area-detector diffractometer	3541 independent reflections
Radiation source: fine-focus sealed tube	1401 reflections with *I* > 2σ(*I*)
graphite	*R*_int_ = 0.078
φ and ω scans	θ_max_ = 25.0°, θ_min_ = 2.1°
Absorption correction: multi-scan (*SADABS*; Bruker, 1998)	*h* = −12→11
*T*_min_ = 0.985, *T*_max_ = 0.991	*k* = −15→15
10293 measured reflections	*l* = −17→15

### Refinement

**Table:**

Refinement on *F*^2^	Primary atom site location: structure-invariant direct methods
Least-squares matrix: full	Secondary atom site location: difference Fourier map
*R*[*F*^2^ > 2σ(*F*^2^)] = 0.055	Hydrogen site location: inferred from neighbouring sites
*wR*(*F*^2^) = 0.185	H-atom parameters constrained
*S* = 1.01	*w* = 1/[σ^2^(*F*_o_^2^) + (0.0662*P*)^2^] where *P* = (*F*_o_^2^ + 2*F*_c_^2^)/3
3541 reflections	(Δ/σ)_max_ < 0.001
273 parameters	Δρ_max_ = 0.18 e Å^−^^3^
0 restraints	Δρ_min_ = −0.19 e Å^−^^3^

### Special details

**Table:**

Geometry. All e.s.d.'s (except the e.s.d. in the dihedral angle between two l.s. planes) are estimated using the full covariance matrix. The cell e.s.d.'s are taken into account individually in the estimation of e.s.d.'s in distances, angles and torsion angles; correlations between e.s.d.'s in cell parameters are only used when they are defined by crystal symmetry. An approximate (isotropic) treatment of cell e.s.d.'s is used for estimating e.s.d.'s involving l.s. planes.
Refinement. Refinement of *F*^2^ against ALL reflections. The weighted *R*-factor *wR* and goodness of fit *S* are based on *F*^2^, conventional *R*-factors *R* are based on *F*, with *F* set to zero for negative *F*^2^. The threshold expression of *F*^2^ > σ(*F*^2^) is used only for calculating *R*-factors(gt) *etc*. and is not relevant to the choice of reflections for refinement. *R*-factors based on *F*^2^ are statistically about twice as large as those based on *F*, and *R*- factors based on ALL data will be even larger.

### Fractional atomic coordinates and isotropic or equivalent isotropic displacement parameters (Å^2^)

**Table:**

	*x*	*y*	*z*	*U*_iso_*/*U*_eq_	
N1	0.1648 (3)	0.5920 (2)	0.2710 (2)	0.0467 (9)	
H1	0.1878	0.5298	0.2818	0.056*	
N2	−0.1766 (5)	0.6049 (4)	−0.0481 (3)	0.0780 (14)	
O1	0.2576 (3)	0.9019 (2)	0.1356 (2)	0.0511 (8)	
O2	−0.1419 (4)	0.5259 (4)	−0.0133 (3)	0.1142 (16)	
O3	−0.2455 (6)	0.6095 (4)	−0.1163 (3)	0.154 (2)	
C1	0.2442 (4)	0.6520 (3)	0.2254 (3)	0.0382 (10)	
C2	0.3791 (4)	0.6138 (3)	0.2166 (3)	0.0473 (11)	
H2A	0.3751	0.5409	0.2050	0.057*	
H2B	0.4301	0.6238	0.2730	0.057*	
C3	0.4497 (4)	0.6650 (3)	0.1429 (3)	0.0455 (11)	
C4	0.4294 (4)	0.7796 (3)	0.1489 (3)	0.0517 (12)	
H4A	0.4839	0.8052	0.1997	0.062*	
H4B	0.4596	0.8109	0.0958	0.062*	
C5	0.2909 (4)	0.8141 (3)	0.1584 (3)	0.0433 (11)	
C6	0.2012 (4)	0.7453 (3)	0.1960 (3)	0.0353 (10)	
C7	0.0640 (4)	0.7834 (3)	0.2070 (3)	0.0372 (10)	
H7	0.0708	0.8533	0.2301	0.045*	
C8	−0.0039 (4)	0.7197 (3)	0.2741 (3)	0.0391 (10)	
C9	−0.1215 (4)	0.7545 (3)	0.3091 (3)	0.0415 (11)	
C10	−0.1783 (4)	0.8501 (3)	0.2861 (3)	0.0493 (12)	
H10	−0.1378	0.8931	0.2474	0.059*	
C11	−0.2924 (4)	0.8803 (4)	0.3201 (3)	0.0644 (14)	
H11	−0.3273	0.9442	0.3057	0.077*	
C12	−0.3568 (5)	0.8154 (5)	0.3765 (4)	0.0744 (16)	
H12	−0.4357	0.8353	0.3978	0.089*	
C13	−0.3039 (5)	0.7236 (4)	0.4000 (3)	0.0653 (15)	
H13	−0.3464	0.6818	0.4387	0.078*	
C14	−0.1868 (4)	0.6900 (4)	0.3676 (3)	0.0520 (12)	
C15	−0.1288 (5)	0.5958 (4)	0.3933 (3)	0.0618 (14)	
H15	−0.1694	0.5544	0.4332	0.074*	
C16	−0.0156 (4)	0.5643 (3)	0.3615 (3)	0.0529 (12)	
H16	0.0207	0.5018	0.3792	0.063*	
C17	0.0466 (4)	0.6269 (3)	0.3013 (3)	0.0423 (11)	
C18	0.5955 (4)	0.6397 (3)	0.1543 (4)	0.0666 (15)	
H18A	0.6306	0.6617	0.2122	0.100*	
H18B	0.6400	0.6742	0.1091	0.100*	
H18C	0.6073	0.5674	0.1488	0.100*	
C19	0.3959 (4)	0.6251 (4)	0.0517 (3)	0.0666 (15)	
H19A	0.4112	0.5530	0.0484	0.100*	
H19B	0.4390	0.6591	0.0055	0.100*	
H19C	0.3037	0.6382	0.0438	0.100*	
C20	−0.0192 (4)	0.7865 (3)	0.1176 (3)	0.0354 (10)	
C21	−0.0601 (4)	0.6960 (3)	0.0759 (3)	0.0404 (11)	
H21	−0.0350	0.6334	0.1009	0.049*	
C22	−0.1379 (4)	0.7000 (4)	−0.0025 (3)	0.0520 (12)	
C23	−0.1796 (4)	0.7900 (5)	−0.0420 (3)	0.0643 (14)	
H23	−0.2344	0.7903	−0.0942	0.077*	
C24	−0.1373 (5)	0.8802 (4)	−0.0016 (3)	0.0660 (15)	
H24	−0.1631	0.9425	−0.0269	0.079*	
C25	−0.0565 (4)	0.8776 (3)	0.0769 (3)	0.0508 (12)	
H25	−0.0267	0.9387	0.1027	0.061*	

### Atomic displacement parameters (Å^2^)

**Table:**

	*U*^11^	*U*^22^	*U*^33^	*U*^12^	*U*^13^	*U*^23^
N1	0.054 (2)	0.034 (2)	0.053 (2)	0.0032 (18)	0.0093 (19)	0.0036 (18)
N2	0.078 (3)	0.089 (4)	0.065 (3)	−0.042 (3)	−0.008 (3)	−0.005 (3)
O1	0.0500 (18)	0.0375 (18)	0.066 (2)	−0.0060 (15)	0.0069 (15)	0.0026 (16)
O2	0.118 (4)	0.078 (3)	0.141 (4)	−0.026 (3)	−0.029 (3)	−0.021 (3)
O3	0.222 (6)	0.148 (4)	0.079 (3)	−0.086 (4)	−0.072 (4)	0.013 (3)
C1	0.036 (3)	0.040 (3)	0.038 (3)	−0.004 (2)	−0.001 (2)	−0.003 (2)
C2	0.045 (3)	0.045 (3)	0.051 (3)	0.005 (2)	0.001 (2)	−0.002 (2)
C3	0.033 (3)	0.051 (3)	0.052 (3)	0.000 (2)	0.004 (2)	−0.001 (2)
C4	0.034 (3)	0.050 (3)	0.072 (3)	0.000 (2)	0.009 (2)	0.004 (3)
C5	0.043 (3)	0.043 (3)	0.043 (3)	−0.005 (2)	0.001 (2)	−0.007 (2)
C6	0.031 (2)	0.035 (2)	0.040 (3)	−0.0017 (19)	0.0066 (19)	−0.002 (2)
C7	0.036 (2)	0.033 (2)	0.042 (3)	0.000 (2)	0.005 (2)	−0.001 (2)
C8	0.038 (3)	0.046 (3)	0.034 (2)	−0.005 (2)	0.002 (2)	0.001 (2)
C9	0.038 (3)	0.051 (3)	0.036 (3)	−0.005 (2)	0.003 (2)	−0.005 (2)
C10	0.045 (3)	0.057 (3)	0.046 (3)	0.002 (2)	0.008 (2)	−0.002 (2)
C11	0.048 (3)	0.082 (4)	0.064 (4)	0.009 (3)	0.009 (3)	−0.010 (3)
C12	0.044 (3)	0.105 (5)	0.076 (4)	−0.003 (3)	0.018 (3)	−0.006 (4)
C13	0.053 (3)	0.090 (4)	0.055 (3)	−0.015 (3)	0.017 (3)	0.002 (3)
C14	0.040 (3)	0.070 (4)	0.047 (3)	−0.010 (3)	0.009 (2)	0.000 (3)
C15	0.068 (4)	0.066 (4)	0.053 (3)	−0.016 (3)	0.016 (3)	0.006 (3)
C16	0.062 (3)	0.049 (3)	0.048 (3)	−0.003 (2)	0.009 (3)	0.010 (2)
C17	0.047 (3)	0.041 (3)	0.040 (3)	−0.005 (2)	0.006 (2)	0.000 (2)
C18	0.041 (3)	0.067 (3)	0.092 (4)	0.005 (2)	0.005 (3)	0.000 (3)
C19	0.069 (4)	0.080 (4)	0.051 (3)	−0.003 (3)	0.010 (3)	−0.007 (3)
C20	0.029 (2)	0.039 (3)	0.038 (2)	0.004 (2)	0.0070 (19)	0.005 (2)
C21	0.036 (3)	0.045 (3)	0.041 (3)	−0.003 (2)	0.005 (2)	0.007 (2)
C22	0.046 (3)	0.072 (4)	0.039 (3)	−0.011 (3)	0.008 (2)	−0.001 (3)
C23	0.055 (3)	0.097 (4)	0.040 (3)	0.000 (3)	0.000 (2)	0.021 (3)
C24	0.070 (4)	0.074 (4)	0.054 (3)	0.024 (3)	0.006 (3)	0.020 (3)
C25	0.054 (3)	0.044 (3)	0.055 (3)	0.002 (2)	0.010 (2)	0.001 (2)

### Geometric parameters (Å, °)

**Table:**

N1—C1	1.355 (5)	C10—H10	0.9300
N1—C17	1.405 (5)	C11—C12	1.401 (6)
N1—H1	0.8600	C11—H11	0.9300
N2—O3	1.201 (5)	C12—C13	1.355 (7)
N2—O2	1.201 (5)	C12—H12	0.9300
N2—C22	1.462 (6)	C13—C14	1.402 (6)
O1—C5	1.241 (5)	C13—H13	0.9300
C1—C6	1.361 (5)	C14—C15	1.411 (6)
C1—C2	1.489 (5)	C15—C16	1.355 (6)
C2—C3	1.524 (5)	C15—H15	0.9300
C2—H2A	0.9700	C16—C17	1.409 (5)
C2—H2B	0.9700	C16—H16	0.9300
C3—C4	1.520 (6)	C18—H18A	0.9600
C3—C18	1.529 (5)	C18—H18B	0.9600
C3—C19	1.529 (6)	C18—H18C	0.9600
C4—C5	1.508 (5)	C19—H19A	0.9600
C4—H4A	0.9700	C19—H19B	0.9600
C4—H4B	0.9700	C19—H19C	0.9600
C5—C6	1.435 (5)	C20—C25	1.381 (5)
C6—C7	1.515 (5)	C20—C21	1.391 (5)
C7—C8	1.518 (5)	C21—C22	1.372 (6)
C7—C20	1.535 (5)	C21—H21	0.9300
C7—H7	0.9800	C22—C23	1.374 (6)
C8—C17	1.371 (5)	C23—C24	1.382 (7)
C8—C9	1.428 (5)	C23—H23	0.9300
C9—C10	1.413 (6)	C24—C25	1.389 (6)
C9—C14	1.423 (6)	C24—H24	0.9300
C10—C11	1.372 (6)	C25—H25	0.9300
			
C1—N1—C17	122.9 (3)	C12—C11—H11	119.8
C1—N1—H1	118.6	C13—C12—C11	119.8 (5)
C17—N1—H1	118.6	C13—C12—H12	120.1
O3—N2—O2	123.3 (5)	C11—C12—H12	120.1
O3—N2—C22	118.6 (6)	C12—C13—C14	121.6 (5)
O2—N2—C22	118.0 (5)	C12—C13—H13	119.2
N1—C1—C6	119.5 (4)	C14—C13—H13	119.2
N1—C1—C2	116.7 (4)	C13—C14—C15	122.3 (5)
C6—C1—C2	123.6 (4)	C13—C14—C9	119.0 (5)
C1—C2—C3	114.4 (3)	C15—C14—C9	118.7 (4)
C1—C2—H2A	108.6	C16—C15—C14	121.7 (4)
C3—C2—H2A	108.6	C16—C15—H15	119.2
C1—C2—H2B	108.6	C14—C15—H15	119.2
C3—C2—H2B	108.6	C15—C16—C17	119.5 (4)
H2A—C2—H2B	107.6	C15—C16—H16	120.3
C4—C3—C2	108.4 (3)	C17—C16—H16	120.3
C4—C3—C18	110.2 (3)	C8—C17—N1	120.5 (4)
C2—C3—C18	109.8 (4)	C8—C17—C16	121.9 (4)
C4—C3—C19	110.5 (4)	N1—C17—C16	117.6 (4)
C2—C3—C19	109.9 (4)	C3—C18—H18A	109.5
C18—C3—C19	108.0 (4)	C3—C18—H18B	109.5
C5—C4—C3	115.9 (3)	H18A—C18—H18B	109.5
C5—C4—H4A	108.3	C3—C18—H18C	109.5
C3—C4—H4A	108.3	H18A—C18—H18C	109.5
C5—C4—H4B	108.3	H18B—C18—H18C	109.5
C3—C4—H4B	108.3	C3—C19—H19A	109.5
H4A—C4—H4B	107.4	C3—C19—H19B	109.5
O1—C5—C6	121.2 (4)	H19A—C19—H19B	109.5
O1—C5—C4	119.6 (4)	C3—C19—H19C	109.5
C6—C5—C4	119.2 (4)	H19A—C19—H19C	109.5
C1—C6—C5	119.3 (4)	H19B—C19—H19C	109.5
C1—C6—C7	122.7 (3)	C25—C20—C21	118.3 (4)
C5—C6—C7	117.8 (4)	C25—C20—C7	121.8 (4)
C6—C7—C8	111.7 (3)	C21—C20—C7	119.9 (4)
C6—C7—C20	111.8 (3)	C22—C21—C20	119.3 (4)
C8—C7—C20	110.0 (3)	C22—C21—H21	120.4
C6—C7—H7	107.7	C20—C21—H21	120.4
C8—C7—H7	107.7	C21—C22—C23	123.0 (5)
C20—C7—H7	107.7	C21—C22—N2	119.2 (5)
C17—C8—C9	119.0 (4)	C23—C22—N2	117.7 (5)
C17—C8—C7	120.2 (4)	C22—C23—C24	117.9 (4)
C9—C8—C7	120.8 (4)	C22—C23—H23	121.1
C10—C9—C14	118.2 (4)	C24—C23—H23	121.1
C10—C9—C8	122.5 (4)	C23—C24—C25	119.9 (5)
C14—C9—C8	119.3 (4)	C23—C24—H24	120.0
C11—C10—C9	120.8 (4)	C25—C24—H24	120.0
C11—C10—H10	119.6	C20—C25—C24	121.6 (4)
C9—C10—H10	119.6	C20—C25—H25	119.2
C10—C11—C12	120.5 (5)	C24—C25—H25	119.2
C10—C11—H11	119.8		
			
C17—N1—C1—C6	−9.7 (6)	C11—C12—C13—C14	−1.5 (8)
C17—N1—C1—C2	165.8 (4)	C12—C13—C14—C15	178.3 (5)
N1—C1—C2—C3	162.1 (3)	C12—C13—C14—C9	0.5 (7)
C6—C1—C2—C3	−22.5 (6)	C10—C9—C14—C13	−0.1 (6)
C1—C2—C3—C4	45.8 (5)	C8—C9—C14—C13	−178.4 (4)
C1—C2—C3—C18	166.2 (4)	C10—C9—C14—C15	−178.0 (4)
C1—C2—C3—C19	−75.1 (5)	C8—C9—C14—C15	3.8 (6)
C2—C3—C4—C5	−47.4 (5)	C13—C14—C15—C16	180.0 (5)
C18—C3—C4—C5	−167.5 (4)	C9—C14—C15—C16	−2.3 (7)
C19—C3—C4—C5	73.1 (5)	C14—C15—C16—C17	0.2 (7)
C3—C4—C5—O1	−157.1 (4)	C9—C8—C17—N1	−177.4 (3)
C3—C4—C5—C6	24.7 (6)	C7—C8—C17—N1	3.1 (6)
N1—C1—C6—C5	171.8 (4)	C9—C8—C17—C16	1.3 (6)
C2—C1—C6—C5	−3.5 (6)	C7—C8—C17—C16	−178.2 (4)
N1—C1—C6—C7	−3.8 (6)	C1—N1—C17—C8	10.1 (6)
C2—C1—C6—C7	−179.0 (4)	C1—N1—C17—C16	−168.7 (4)
O1—C5—C6—C1	−175.7 (4)	C15—C16—C17—C8	0.3 (7)
C4—C5—C6—C1	2.5 (6)	C15—C16—C17—N1	179.0 (4)
O1—C5—C6—C7	0.1 (6)	C6—C7—C20—C25	−109.8 (4)
C4—C5—C6—C7	178.2 (4)	C8—C7—C20—C25	125.4 (4)
C1—C6—C7—C8	15.0 (5)	C6—C7—C20—C21	70.8 (4)
C5—C6—C7—C8	−160.6 (3)	C8—C7—C20—C21	−53.9 (5)
C1—C6—C7—C20	−108.8 (4)	C25—C20—C21—C22	−1.5 (6)
C5—C6—C7—C20	75.6 (4)	C7—C20—C21—C22	177.9 (4)
C6—C7—C8—C17	−14.3 (5)	C20—C21—C22—C23	−0.9 (6)
C20—C7—C8—C17	110.5 (4)	C20—C21—C22—N2	177.6 (4)
C6—C7—C8—C9	166.3 (3)	O3—N2—C22—C21	179.4 (5)
C20—C7—C8—C9	−68.9 (4)	O2—N2—C22—C21	2.4 (7)
C17—C8—C9—C10	178.5 (4)	O3—N2—C22—C23	−2.0 (7)
C7—C8—C9—C10	−2.0 (6)	O2—N2—C22—C23	−179.0 (5)
C17—C8—C9—C14	−3.3 (6)	C21—C22—C23—C24	2.0 (7)
C7—C8—C9—C14	176.2 (4)	N2—C22—C23—C24	−176.6 (4)
C14—C9—C10—C11	0.8 (6)	C22—C23—C24—C25	−0.6 (7)
C8—C9—C10—C11	179.0 (4)	C21—C20—C25—C24	2.8 (6)
C9—C10—C11—C12	−1.8 (7)	C7—C20—C25—C24	−176.6 (4)
C10—C11—C12—C13	2.2 (8)	C23—C24—C25—C20	−1.7 (7)

### Hydrogen-bond geometry (Å, °)

**Table:**

*D*—H···*A*	*D*—H	H···*A*	*D*···*A*	*D*—H···*A*
N1—H1···O1^i^	0.86	2.13	2.937 (4)	156

Symmetry codes: (i) −*x*+1/2, *y*−1/2, −*z*+1/2.
